# Supplementary material for: A novel missense variant in the ATPase domain of ATP8A2 and review of phenotypic variability of ATP8A2-related disorders caused by missense changes
Source: Neurogenetics. 2024 Jul 27;25(4):425–33. doi: 10.1007/s10048-024-00773-9 (PMC11534842; doi:10.1007/s10048-024-00773-9)
Supplement: Supplementary file 1 — Supplementary Material 1 [file 10048_2024_773_MOESM1_ESM.docx]

| **ACMG Evidence Class** | **Description** | **Justification for this Case** |
| --- | --- | --- |
| PS3 (Strong) | Well established *in vitro* or *in vivo* functional studies supportive of a damaging effect on the gene or gene product. | The p.Leu538Pro variant causes nearly complete loss in ATP8A2 expression. |
| PM1 (Moderate) | Located in a mutational hotspot and/or critical and well-established functional domain (e.g. active site of an enzyme) without benign variation. | Our cases and others confirm that the catalytic cytoplasmic N and P domains are mutational hotspots. |
| PM2 (Moderate) | Absent from controls (or at extremely low frequency if recessive) in the Genome Aggregation Database. | The L538P variant is absent from the Genome Aggregation Database. |
| PP2 (Supporting) | Missense variant in a gene that has a low rate of benign missense variation and in which missense variants are a common mechanism of disease. | There are 185 missense variants in ATP8A2 reported in ClinVar, of which 6 are curated benign and/or likely benign. **Date Accessed: 18 June 2024** |
| PP3 (Supporting) | Multiple lines of computational evidence support a deleterious effect on the gene or gene product (conservation, evolutionary, splicing impact, etc.). | SIFT, PolyPhen, CADD, REVEL, and FoldX5 all support a deleterious effect of L538P on ATP8A2. |

**Supplementary Table 1. ACMG criteria supporting the classification of the p.Leu538Pro variant as likely pathogenic.**
